# Reviews

**Published:** 1874-12

**Authors:** 


### REVIEWS.

I find on my table the Annual Report of the Baltimore Eye and Ear Institute, (from the Surgeon-in-charge,) Julian J. Chisolm, M. D. This report contains many interesting cases of diseases of the eye and ear, such as pariphemoses of the eyelids; suppurative iritis; lenticular opacity, etc., of the eye; and of the ear, such as rupture of the drum, acute inflammation of the middle ear, etc., with a history of a case of deafness of three years duration promptly relieved by the use of the Eustachean catheter.

A pamphlet from the Author (Wm. E. Dabney, M. D., Charlottsville, Va.) The value of Chemistry to the Medical Practitioner. This is a most valuable production, and reflects great credit upon the author. It should be in the possession of every practitioner of medicine. I will refer to this pamphlet again.

A pamphlet from the Author, (Hunter McGuire, M. D., Professor of Surgery in the Medical College of Virginia, Richmond,Va.)

containing interesting cases of gun-shot wounds of the peritoneum, and showing the importance of *drainage* in these wounds. These cases reported by my friend Dr. McGuire are exceedingly interesting, and are possessed of peculiar interest and value to the physician and surgeon. These cases, as reported, are plain, practical and to the point.

A Circular, announcing a forthcoming History of the North-American Birds, by Spencer F. Baird, M. D., Thomas M. Brewer, and Robert Ridgway. Professor Baird's high reputation as a gentleman of scientific attainments, is a sufficient guarantee that the forthcoming work will be of great value to all lovers of "ornithological" lore.

A pamphlet on Fish Culture, by Middleton Goldsmith, M. D., Commissioner on Fish Culture for the State of Vermont. We know the Doctor well: besides being eminent as a physician and surgeon, what he does not know of *wood*-craft, from breaking a setter, to "landing a three-pound trout," is not worth knowing.

Columbia Hospital for Women; report by J. H. Thompson, with department of diseases of Children, by Samuel C. Busy, M. D.; section of diseases of the Eye and Ear, by D. Webster Prentiss, M. D., and report of the section of "Diseases of Females" at Columbia Hospital Dispensary, by F. A. Ashford, M. D. I am under many obligations to Dr. Ashford for this truly valuable work. I will recur to this work hereafter, as I consider it replete with *recent* information.

Transactions of the Fourth Annual Session of the Medical Society of Virginia, 1873, contains a valuable series of medical papers. It is highly creditable to the Socie.y. The address of the retiring President, Dr. Harvey Black, is a sound, able paper, well worthy of the high standing and character of the man. The annual address to the public and profession, on the reciprocal relations of the medical profession and communities, by R. S. Hamilton, M. D., is a very sensible and truly eloquent production.

Supplimental Report, to the one presented at the third session, on the Anatomical, Physiological and Pathological differences between the White and the Black Races, by Thomas P. Atkinson,

M.D., Amelia county, Va., is possessed of rare merit, proving its author to be capable ef vast research and *semper parotus* to deal quick, heavy blows with a sharp, polished rapier. I rather think my old and highly esteemed friend put his blade under the fifth *rib* of his Bostonian reviewers. Glad to see he retains some of the fire of youth.

Some remarks on Intermittent and Remittent Fevers, suggested by their recent prevalence in Louisa county, by Wm. A. Gillespie, M.D. Dr. Gillespie shows an original turn of thought and good discriminating judgment in the treatment of his cases.

Nitrate Amyl as an Antidote to Chloroform. By Wm. C. Dabney, M.D. Dr. Dabney's experiments are so exceedingly interesting, that I hope the Doctor may be induced to continue his experiments still further, and to give us his experience. These experiments recall to my mind the fact of my having given a small dog once a ʒi dose of strychnia, and by repeatedly administering chloroform immediately and as often as the "spasms" gave signs of returning, staved off death some six or eight hours, until we became satisfied that death could be prevented indefinitely. The dog would suck the chloroform with all the avidity of a young hungry calf sucking its mother. I am not, however, prepared to say whether chloroform possesses any antidotal powers versus the alkaloid of nux vomica, or only averts the "tendency to death" therefrom, by counteracting and controlling spasmodic action.

Chloroform in Obstetrical Practice. By ex-President A. M. Fauntleroy, Staunton, Va. This valuable paper gave rise to a long and able debate, participated in by Drs. John H. Claiborne, Hunter McQuire, George A. Forte, (Warrenton, N. C.,) Wm. L. Brown, T. J Riddell, ex-President R. S. Payne and O. B. Jenks. This long discussion was marked by ability on both sides of this question.

A short and clever report on Epidemics of Piedmont District. By D. A. Langhome, M.D., Chairman. My distinguished colleague, Dr. F. Horner, Jr., in this report states that rubeola and scarlatina has been both rare and mild; that an epidemic of influenza followed the epizooty in that county; "and that *inebriety*, claimed by some to be a disease, was common, and at one time was so universal in some localities, as to prove most destructive to the health and well-being of the community." Was not this influenza the epizooty itself? The exuberance of feeling, incident to health and youth, I have known mistaken for inebriety. I would suggest to my estimable colleagues that this *disease* does not occur in my section of the country, where the people are furnished with a better example to follow. I know my friend is a gentleman of medical acumen, and I regret that he has permitted his enthusiasm in the cause of temperance to mislead his judgment, and to do an unintentional injustice to the citizens of his native county. I know his motives were pure, however. But when we remember the four years of terrible warfare through which these people passed, we can only wonder at the small amount of demoralization manifested by the citizens of the South generally. For intelligence, *sobriety* and thrift, the male population of old Fauquier county will compare favorably with the citizens of any other county in this State, or any other State in this Union; whilst her daughters, for all the noble attributes that go to adorn and make lovable the female character, they are second to none in the United States or the Canadas. I repeat, I regret this oversight, for it mars this otherwise capital report.

Remarkable Recovery from a Pistol-shot Wound of the Right Illium: Pelvis Trephined to drain Pyemic Secretions. The case is easily treated and handsomely reported by First Vice-President A. E. Gleaves, Wytheville, Virginia.

Saccharated Pepsin. By C. Wortley Thomas, Hyde county, North Carolina. This paper will well pay careful perusal. A wound of the pelvis, and the value of Nelaton's Porcelain Prob exemplified by William G. Semple, M.D., Hampton, Virginia.

Some Neglected Points in the Pathology of Typhoid Fever, as furnishing the proper indications of Treatment. By Samuel K. Jackson, M.D., Norfolk, Virginia. It is seldom that I have read anything that has afforded me more pleasure than I have experienced by the perusal of this valuable little *troussedu* from the pen of my good friend, Dr. S. K. Jackson. Yet I hope he will pardon me for entering a demurrer to some of his deductions and conclusions. He says: "I know but little of the history of typhoid fever anterior to the beginning of my professional career in 1839; yet am unwilling to admit with Dr. Payne, in his very interesting account of it in the very region in which I first made its acquaintance, that it was confined to any particular locality, or had made its appearance at any particular time; but am inclined to the opinion that it existed for an indefinite period over all that portion of the continent, where malarious fevers did not prevail." During the late war I received a summons from General Blenker to appear before him, and to testify as to the danger of attempting to cross the Shenandoah river with his army. When I arrived, the General said: "Dr. Payne, I learn that you are an old resident of this section of the country and an observant man. I want you to tell me all about the Shenandoah river." My reply was: "I hope General Blenker will remember that for all this length of time my mind has been on *medicine*, not on the Shenandoah river." I wish my friend Dr. Jackson also to remember that I was engaged for over twenty years in endeavoring truthfully to locate the inception of typhoid fever in Piedmont, Va., both as to time and locality. All this time his mind was probably engaged upon other subjects. I repeat, if there is any reliability in human testimony, I am correct when I say it commenced about the year 1835, in the family of Mr. J. Diskin Ferguson, who lived in Clark county, west of Blue Ridge, and between Berry's Ferry, on the Shenandoah river, and the Blue Ridge Mountains (a narrow strip of country); that it ravaged this narrow strip of country for several years before it pushed the head of its column over the Blue Ridge and invaded that beautiful and fertile region of upper Fauquier and Loudoun counties, and where Dr. Jackson first encountered it. "But am inclined to the opinion that it existed for an indefinite period over all that portion of the continent where malarious fevers did not prevail." This is rather ambiguous, and must mean that all this continent, at all times, is bound to have either a fever of a malarious or non-malarious origin prevailing within its boundaries; or the Doctor supports the hypothesis that continued fevers follows those of a malarial type. Now, it is a well-established fact that in new countries where the primeval forest, the deep lake, the dark lagoon, and the broad marsh exist, there the malarial type of fever prevail to the greatest extent, force and power. Therefore if these malarial fevers are naturally followed by thoss of a remtitent and continued type, it follows that Dr. Jackson believes that, by cultivation, drainage, etc., you rather destroy and than improve the health of a country. Does the Doctor mean to stand *squarely* by this hypothesis?

The Doctor further says: "Nor can I agree with Dr. Payne in considering any one in that or any other region, and at that day, as 'mastering the situation.'" This is a fair hit. I cheerfully acknowledge the corn. I should have said "improved the situation;" and I should have said so, but for a reluctance I felt to claim the honor of being the first to administer large doses of quinine and other stimulants in typhoid fever in the Piedmont region of Va. I learned this plan of treatment from the teachings of Wm. H. Van Buren, son-in-law of the great Valentine Mott, M.D., then Physician and Surgeon to Bellevue Hospital, afterwards a distinguished Professor in the Medical University of New York City. I cannot be reasonably accused of favoring the "Expectant" plan of treatment, either in the administration of medicine or of diet, for I recommend heavy doses of stimulants, with beef-tea, butter milk, etc., *ad libitum*. I also say, in time, it pays its visit to all; calls alike at the filthy hovel and at the cleanly palace; invades the rugged mountain top, and falls with frightful power upon the inhabitants of the green and smiling valley. But the only real, apparent, important difference between Dr. Jackson, in treating typhoid fever, and myself, will be found in the fact that whilst I always am glad to put my patients on the "alcoholic stimulants," as soon as palatable to them, he entirely ignores their use in every stage of the disease. The correctness of my pathology, *i. e.* ulceration of the glands of Peyer and of Bruns, for typhoid fever, has been proven by myself in over a hundred post-mortem examination of the cadaver; besides being corroborated by all the ablest pathologists in Germany, France, England and America. I would not encumber great pathological truths, well understood and well recognized by many pathologists in different climes, with "neglected points in its pathology"—only recognized by a learned few, and disputed by a majority of the profession in Europe and America. The believers in the "coloric test for typhoid fever" admit the correctness of my pathology.

Hear what Drs. Latham and Brittain have to say on this subject. When is a patient convalescent from typhoid fever? is a question thus answered by that excellent observer, Dr. P. W. Latham: "The only satisfactory answer, in my opinion, is after the morning and evening temperatures, especially the latter, on at least two successive days have remained about the normal point, or between 98° and 99° F." In Dr. Brittain's case, the temperatures varied from 98° to 100°, not being higher than 100.6°. The daily variations are not given, but it would be instructive to know whether, before the collapse on the 23d, the patient's temperature on at least two succesive days, at 8 A.M. and 7 P.M., had remained between 98° and 99°. Two years ago, I referred to cases where, after all the general symptoms of typhoid fever had passed away, considerable hemorrhage took place from the bowels; and I also stated that during the period of defervescence, the patient's tongue may be clean and moist, the appetite ravenous, the patient "crying out" for food, and the typhoid ulcer still unhealed. The thermometer alone will tell us this. It will probably show at this stage an evening temperature of about 101° F., with a morning temperature of 1.6° to 2° lower, and a mutton-chop might now be sufficient to induce fresh irritation of the intestinal ulcers, fatal hemorrhage or perforation. It is only after the evening temperature has remained *on at least two successive days* below 99° F., that we can be sure the ulcers have healed, and that solid food may be given without risk. I have never seen a relapse or hemorrhage supervene after a patient was *really* ravenous for food—so ravenous that he would eat a "mink" skin, and all if he had one. I doubt whether this "*ravenous*" appetite occurs before the evening temperature is about 98° F. If I was asked when I considered a patient convalescent from typhoid fever, my answer would be: After he eats well, drinks well and sleeps well, and has continued to do so for two or more successive days. I hope Dr. Jackson will favor us with a fuller and more lengthy article on this subject. No one will be more pleased to read his valuable contributions to science than myself.

Reports of Two Cases: 1. Foreign Body in the Rectum; 2. Hepatic Abcess. By Wm. H. Bramlettee, Newburn, Pulaski county. These cases are interesting to the practitioner of medicine, and are related in an agreeable, sensible manner.

The subject for discussion was cerebro-spinal meningitis. Dr. T. J. Riddell opened the discussion by reading a well-prepared paper on this disease. The discussion was continued by Drs. L. B. Edwards and W. W. Parker, both of whom acquitted themselves handsomely. A. S. P.

